# Diagnostic Value of Measurement Specific Gravity by Refractometric and Dipstick Method in Differentiation between Transudate and Exudate in Pleural and Peritoneal Fluid

**Published:** 2016

**Authors:** Alireza Abdollahi, Zohreh Nozarian

**Affiliations:** *Dept. of Pathology, Imam Khomeini Hospital Complex, Tehran University of Medical Sciences, Tehran, Iran*

**Keywords:** Specific Gravity, Refractometry, Dipstick, Transudate, Exudate, Pleural and Peritoneal Fluid

## Abstract

**Background:**

Accumulation of pleural and peritoneal fluid is seen in some diseases. In order to diagnose the disease and start the treatment, one of the most important actions will be to differentiate between exudates and transudates. The objective of this study was to determine the diagnostic value of measuring the specific gravity of the fluid through refractometer and strip in differentiation of exudates from transudates.

**Methods:**

The serum of patients was evaluated for protein, LDH, cholesterol, bilirubin and albumin. The fluid was evaluated for the number of white blood cells, protein, LDH, cholesterol, bilirubin and albumin. Then the fluids were divided into exduate and transudate categories based on Light and Gradient criteria. Finally, the specific gravity of the fluids was measured by refractometer, Erma, Japan and Medi-Test Combi II. The categorized fluids were compared with Gold Standards (final diagnosis) so that the sensitivity and specificity of Light and Gradient criteria in the transudate-exudate differentiation were specified.

**Results:**

In comparison with Light criteria, the cut off level of 1022 specific gravity measured by refractometer for pleural effusion has sensitivity, specificity of 92.1%, 68.1%respectively. In evaluation of peritoneal fluid considering cut off point 1023, measured by refractometer has reliable sensitivity 92.4%, specificity 70.4 compared with standard gradient method.

**Conclusion:**

Differentiating transudate from exudates by measuring its special gravity by refractometer will have acceptable sensitivity and specificity, and when rapidity is necessary or access to lab equipment is limited, this method could be used.

## Introduction

Accumulation of pleural and peritoneal fluid is seen in some diseases (1-3). Exudative effusions seen in some diseases such as lupus, rheumatoid arthritis, Wegener, asbestosis, sarcoidosis, uremia and transudate effusions seen in heart failure, nephrotic syndrome, hypoalbuminemia and etc.

 In order to diagnose the disease and start the treatment, one of the most important actions will be to differentiate between exudates and transudates (3-5). Light et al. proposed a method for differentiation between exudates and transudates in 1972 accepted so far (4-11). The sensitivity and specificity of this criterion is respectively 99 and 98 percent (6). In Light’s criteria, the ratio of protein to serum in the fluid is below 0.5, the pleural fluid’s cholesterol is less than 60 mg/dl and the ratio of cholesterol to serum is below 0.3 in favor of tansudativity, while the ratio of protein to serum is above 0.5, the pleural fluid’s cholesterol is above 60 mg/dl and the ratio of cholesterol to serum is above 0.3 in favor of exudativity (7-12).

As far as the peritoneal fluid is concerned, the gradient of albumin level of serum to fluid is above 1.1 in favor of transudativity and less than 1.1 in favor of exudativity (12-18). Measurement and calculation of these analytes require time and equipment which may not be accessible everywhere and treatment may be delayed and consequently mortality and morbidity will increase. Measurement of the fluid’s specific gravity has been proposed for differentiating exudates from transudates, but due to differences of opinion, this method is rarely applied (18-21). This method is also less costly than other methods. 

The objective of this study was to determine the sensitivity and specificity of measuring the specific gravity of the fluid through refractometer in differentiation of exudates from transudates and comparing it with the specific gravity measured by Medi-Test Combi II.

## Materials and Methods

This prospective and cross-sectional study was conducted at Imam Teaching Hospital, Tehran University of Medical Sciences, Tehran, Iran within 2013-2014. This Hospital is an education, research and healthcare center affiliated with Tehran University of Medical Sciences. All pleural and peritoneal fluids delivered from different wards were included in the study. The coagulated fluids lacked blood samples excluded. The blood samples with fluid were centrifuged for 15 min at 3000 g speed and the serum obtained from this process was evaluated in terms of amount of protein, LDH, cholesterol, bilirubin and albumin. The fluid was also evaluated after macroscopic studies (for the presence or absence of clots, color, transparency and volume) in terms of the number of white blood cells, protein, LDH, cholesterol, bilirubin and albumin.

The analytes were evaluated by BT-3500, Italy auto-analyzer and with biosystem, Spain kits. The quantitative measurement of protein was done with Biuret photometric method, the LDH with DGKC photometric method, bilirubin with DCA photometric method, albumin with Bromocresd green photometric method and cholesterol with CHOD-PAP photometric method.

After the amount of analytes was known, the fluids were divided into exudate and transudate categories based on Light and Gradient criteria. Then the specific gravity of the fluids was measured by refractometer, Erma, Japan and Medi-Test Combi II.

The categorized fluids were compared with Gold Standards (final diagnosis) so that the sensitivity and specificity of Light and Gradient criteria in the transudate-exudate differentiation were specified. Then, the sensitivity and specificity of measurement of specific gravity of fluids was done with two methods – refractometer so that the strip in the exudate-transudate differentiation was determined and compared with the Light's criteria and albumin Gradient concentration.

The Ethics Committee of Tehran University of Medical Sciences approved the study protocol according to the declaration of Helsinki. All participants were given information regarding their consents before entering the study. Our gathered data were confidential and no extra cost was constrained on our participants.

**Statistical analysis**

The usefulness of each biochemical parameter and other calculations for identifying exudates was evaluated using Bayesian methods to measure the following: sensitivity, TP/ (TP+FN); specificity TN/ (TN+FP); accuracy (TP+TN)/ (TP+TN+FP+FN); Positive predictive value, TP/TP+FP; Negative predictive value, TN/TN+FN where TP is the number of true positive diagnoses, FP; the number of false positive diagnoses, TN; the number of true negative diagnoses, and FN; the number of false negative diagnoses. These indices were compared using mcnemar's exact test for correlated proportions.

Descriptive indices such as frequency, percentage, mean and standard deviation (SD) were used to express data. All analyses were done using SPSS software version 13.0 (SPSS Inc, Chicago, IL). Significance level was defined as *P*<0.05.

## Results

Out of total 268 fluids delivered, 125 were pleural and 143 were peritoneal. Of the 125 pleural fluids, based on Light criteria, 61 cases (49 men and 12 women) were exudates and 64 (40 men and 24 women) were transudates. Of the 143 peritoneal fluids, based on albumin gradient measurement, 65 cases (51 men and 14 women) were exudates and 78 (48 men and 30 women) were transudates (Table1).

Tables 2 and 3 respectively show the causes of exudation and transudation like malignancies, congestive heart failure, empyema etc., in pleural and peritoneal fluids. Tables 4 and 5 respectively show the sensitivity, specificity, PPV and NPV, measured by refractometer and medi-test, combi II in pleural fluids at different cut-off points.

**Table 1:** Characteristics of body fluids

Total : 268 Fluids			
Peritoneal Fluid 143 (53.3%)		Pleural Fluid 125 (46.6%)	
Transudative	Exudative	Transudative	Exudative
78 (54.5%)	65 (45.4%)	64 (51.2%)	61 (48.8%)

Tables 6 and 7 show respectively the sensitivity and specificity, PPV, NPV, measured by refractometer and medi-test, combi II in peritoneal fluids at different cut-offs. 

Sensitivity, specificity, PPV, NPV, measurement of specific gravity by refractometer in the exudative, transudative differentiation of pleural and peritoneal fluids is much better than the medi-test, combi II methods. 

**Table 2:** Cause of transudative and exudative pleural effusions

**Cause**	**Exudative (%)**	**Transudative (%)**	**Total (%)**
Malignancies	41(67.2)	-	41(32.8)
Congestive heart failure	-	36(56.2)	36(28.8)
Empyema	5(8.1)	-	5(4)
Tuberclosis	4(6.5)	-	4(3.2)
Pulmonary embolism	2(2.9)	3(4.6)	5(4)
End stage renal disease	-	6(9.3)	6(4.8)
Pnemonia	9(14.7)	-	9(7.2)
Nephrotic syndrome	-	4(6.2)	4(3.2)
Lupus erythematous	-	2(3.1)	2(1.6)
Unknown	8(13.1)	10(15.6)	18(14.4)
Hepatic cirrhosis	3(2.4)	3(4.6)	-

**Table 3:** Cause of transudative and exudative peritoneal fluids

**Cause**	**Exudative (%)**	**Transudative (%)**	**Total (%)**
Cirrhosis	-	50(64.1)	50(34.9)
Heart failure	-	9(11.5)	9(6.2)
Constrictive pericarditis	-	2(2.5)	2(1.3)
Nephrotic syndrome	-	5(6.4)	5(3.4)
Kwashiorkor	-	1(1.2)	1(0 /6)
Malignancies	49(75.3)	-	49(34.2)
Tuberclosis	3(4.6)	-	3(2)
Spontaneous bacterial peritonitis	4(6.1)	-	4(2.7)
Pancreatitis	2(3)	-	2(1.3)
Unknown	7(10.7)	11(14.1)	18(12.5)

**Table 4:** Sensitivity, Specificity, Positive and Negative predictive values at different specific gravity cut-off values measured by refractometer (pleural fluid

NPV (%)	PPV (%)	Specificity (%)	Sensitivity (%)	Specific gravity
81.3	82.1	37.3	96.1	1018
79.1	83.8	38.9	95.3	1019
78.9	84.6	43.1	94.1	1020
78.9	86.3	57.3	93.8	1021
78.3	88.1	68.1	92.1	1022
76.6	89.2	69.4	92	1023
75.1	90.8	76.1	91.1	1024
74.2	91	83.3	89.8	1025
73.3	91.9	91.2	88.6	1026

**Table 5.** Sensitivity, Specificity, Positive and Negative predictive values at different specific gravity cut-off values measured by medi-test, combi II (pleural fluid

NPV (%)	PPV (%)	Specificity (%)	Sensitivity (%)	Specific gravity
71.3	75.1	41.3	88	1020
70.4	76.8	49.2	87.6	1021
69.9	77.3	58.4	87.3	1022
69.1	78.2	65.1	86.3	1023
68.2	79.3	66.3	84.6	1024
67.3	79.6	66.8	83.8	1025
66.4	78.1	67.2	82.9	1026

**Table 6:** Sensitivity, Specificity, Positive and Negative predictive values at different specific gravity cut-off values measured by refractometer (peritoneal fluid

NPV (%)	PPV (%)	Specificity (%)	Sensitivity (%)	Specific gravity
77.2	78.6	47.1	94.6	1020
77.9	82.3	59.3	93.3	1021
78.1	89.1	63.1	92.5	1022
78.5	85.2	70.4	92.4	1023
77.1	89.8	74.1	90.1	1024
76.2	90.6	80.3	89.1	1025
75.1	91.3	84.2	88.4	1026

**Table 7.** Sensitivity, Specificity, Positive and Negative predictive values at different specific gravity cut-off values measured by medi-test, combi II (peritoneal fluid

NPV (%)	PPV (%)	Specificity (%)	Sensitivity (%)	Specific gravity
72.1	75.5	44.1	87.1	1020
71.7	75.2	49.6	86.6	1021
69.9	74.3	61.4	85.3	1022
67.1	69.2	63.1	84.3	1023
67.9	74.3	65.6	84.1	1024
66.9	76.6	66.3	82.6	1025
65.1	78.8	66.9	81.4	1026

## Discussion

There are many causes of pleural and peritoneal effusion and correct diagnosis of the underlying disease is a major challenge for clinicians and essential to correct patient management. Separation of exudates from transudate remains a useful initial step in determining the cause of an effusion. A transuda result from an imbalance of starling forces ending to movement of fluid into the serosal spaces with little amount of proteins or other large molecules but exudative effusion results from serosal surface disease and is rich in protein, inflammatory cells and mediator (1-5).

The management of pleural and peritoneal effusion is still a difficult clinical problem and misclassifications were made resulting in patient enduring unnecessary investigation (6-9).

Some studies considered the CRP level of pleural liquid useful in differentiating transudate from exudates (19-21). The viscosity of the pleural liquid in exudative materials is higher than in transudative liquids. Measurement of viscosity for differentiation is recomended (22).

A combination of multiple parameters was used by Light et al in 1972. Lightś criteria with high sensitivity and specificity are used for differentiating exudates from transudate in pleural fluid (5-11, 20).

In peritoneal fluids, acceptable item is the gradient for albumin level serum to fluid for this differentiation, but evaluation of these analytes require time, equipment and measurement of multiple analyte that may not be accessible and due to delay in diagnosis, treatment and consequently increased mortality and morbidity (17-21) .

In our study, we measured the fluid specific gravity (SG) with refractometer and dipstick medi-test, combi II. The refractive index and the specific gravity of liquids depended upon the total solid dissolved in the fluid .We have found that pleural and peritoneal specific gravity of the patients with transuda was significantly lower than of patient with exudative accumulation .

We compared our results in both methods with the most acceptable sensitive and specific light and gradient criteria. According to our study on specific gravity, pleural fluid in exudative effusion was significantly greater than to transude.

The cut-off level of 1022 for pleural effusion has sensitivity, specificity, PPV and NPV of 92.1%, 68.1%, 88.1% and 78.3% respectively. This cut-off point yielded the best results in differentiating exudates from transudates. Although specific gravity of 1018 has a good sensitivity (96.1%) but low specificity in this cut-off can elevate false positive results. In same cut-off level, 1022, which was measured by dipstick method had a low sensitivity of (87.3%), specificity (58.4%), PPV (77.3%) and NPV (69.9%) which are not acceptable in comparison with standard lights criteria. In addition, selecting cut-off 1018 with higher sensitivity in dipstick method due to less specificity is not recommended.

Regarding the evaluation of peritoneal fluid considering cut-off point of 1023 measured by refractometer has reliable sensitivity (92.4%), specificity (70.4), PPV (85.2%) and NPV (78.5%) in comparison with standard gradient method. However, as mentioned in pleural fluid specific gravity by dipstick method had no acceptable results and this modality is not recommended for distinguishing transudate from exudates in peritoneal and pleural fluids.

In this study, specific gravity of exudative effusion was higher than that of transudative effusion with high sensitivity, specificity, PPV, and NPV in comparison with standard methods. Regarding simplicity, costly and rapidity of this measurement may play a valuable role in the accurate and fast discrimination of serosal fluid.

Restrictions to the study include the low volume of samples and lack of body liquids including joint or pericardial liquids. Search engines provided very few links to similar studies and therefore a comparison was impossible. Therefore, a study with more samples is recommended in several centers with several body liquids.

## Conclusion

Differentiating transudate from exudates by measuring their special weights using refractometer will have acceptable sensitivity and specificity, and when rapidity is necessary or access to laboratory equipment is limited, this method could be used. Measuring the special weight by strip is an easy and quick method, but has lower specificity than refractometer. Moreover, there are different marks of strips and therefore this method is not recommended.
